# Relaxation behavior of polyethylene welded joints

**DOI:** 10.1186/s11671-017-2059-z

**Published:** 2017-04-18

**Authors:** Valeriy Demchenko, Maksym Iurzhenko, Andriy Shadrin, Anatoliy Galchun

**Affiliations:** 1 Plastics Welding Department, E.O.Paton Electric Welding Institute, National Academy of Sciences of Ukraine, B.8, 11, Kazymyra Malevycha Str., 03680 Kyiv-150, Ukraine; 2 Institute of Macromolecular Chemistry, National Academy of Sciences of Ukraine, 48 Kharkivske Av., 02160 Kiev, Ukraine

**Keywords:** Polyethylene, PE-80, PE-100, Butt fusion, Welding, Relaxation, 81.20.Vj, 81.05.Lg, 81.07.-b

## Abstract

The paper presents results of the investigation of structure relaxation and thermal properties of PE-80 and PE-100 polyethylene hot-tool butt welds. It was found that a weld with the re-crystallized crystalline structure is formed during the welding of dissimilar types of polyethylene. It is shown that within a long period (1 year) the relaxation occurs not only in amorphous but also in the crystalline phase (crystalline α-form transforms into mixed αβ-form), with respective changes in polyethylene properties.

## Background

Progress in the modern material science has caused wide expansion of thermoplastics application in various industries: chemical, construction, medical, radio-technical, electronic, foodstuff etc. [1–6].

Welding process for thermoplastic polymers happens with activation of welded surfaces either before bringing these surfaces in contact (hot tool, hot gas, or IR-radiation welding) or with activation of surfaces simultaneous with bringing surfaces in contact (friction or ultrasonic welding) [7].

Along the cooling of joint, the super-molecular structure is formed in the weld; such welds have the respective stress fields which are relaxing with time [8]. These competing processes determine the final properties of the welded joints. The technological goal is to receive the joint with the properties as close as possible to the properties of the parent raw material.

Various physical and chemical transformations occur in the joint—melt fluidity is changing, orientation crystallization, re-crystallization, and even partial destruction occur, and as a result we receive heterogeneous structure of the welded joint [9].

Thereby, the goal of this work is a complex investigation of structure and property relaxation behavior in welded joints of dissimilar types of polyethylene, using the methods of wide- and small-angle X-ray scattering, differential scanning calorimetry, and thermal analysis.

## Methods

### Materials and processing

The following two types of high-density polyethylene (HDPE) specimens with different long-term minimum required strength (MRS, within 50 years at 20 °C) have been used for welding experiments and further structural, thermal, and field performance data investigations: PE-80 (MW_bimodal_ 300,000 g/mol, density 0.953 g/cm^3^, MRS 8 MPa) and PE-100 (MW_bimodal_ 300,000 g/mol, density 0.960 g/cm^3^, MRS 10 MPa).

**Fig. 1:**
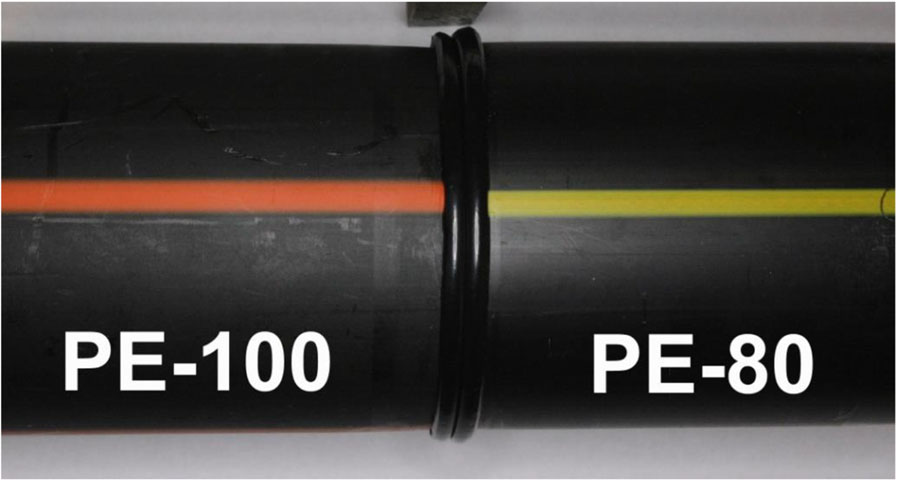
Welded joint of PE-80 and PE-100 polyethylene pipe types

Hot-tool butt welding experiments have been carried out on pipes with 63-mm outside diameter and 6-mm wall thickness, with the following welding parameters: hot tool temperature 200 °C, upsetting pressure 0.2 MPa within 60 s, with dwell time 3 s and cooling time 6 min. Serial SAT-1 hot-tool butt welding device produced by E.O.Paton Electric Welding Institute’s factory has been used for the welding experiments. Figure 1 represents the photo of PE-80 with PE-100 welded joint.

### Equipment and measurements

**Fig. 2:**
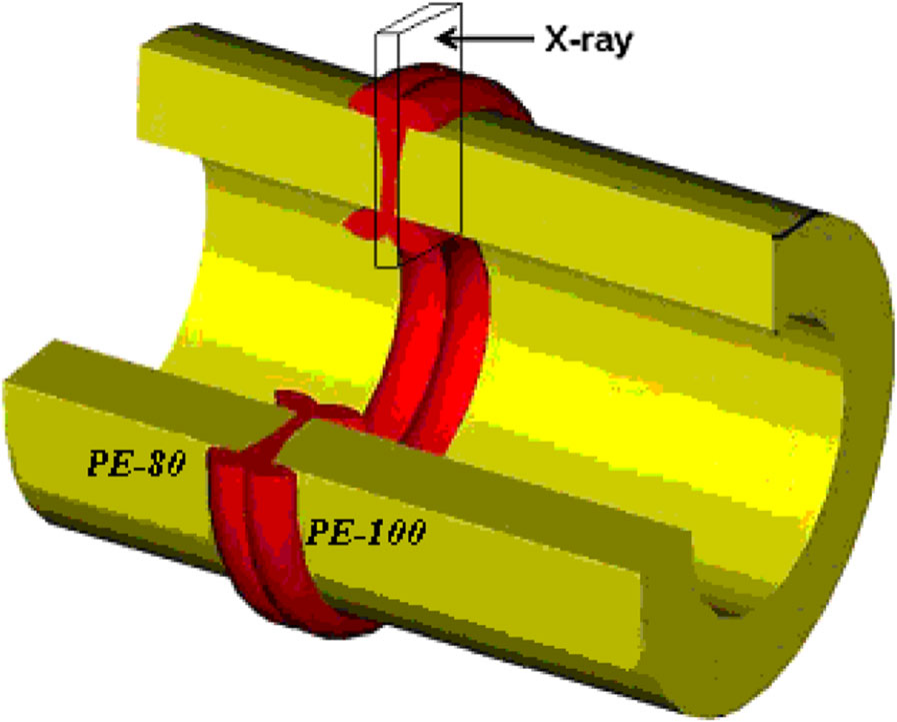
Scheme of welded joint of PE-80/PE-100 polyethylene pipe types with position of a sample for X-ray study

The amorphous and amorphous-crystalline structure peculiarities of PE-80 and PE-100 specimens, as well as PE-80/PE-100 welds (Fig. 2) have been investigated by wide-angle X-ray scattering (WAXS) method using DRON-4-07 diffractometer (Burevestnik, Saint Petersburg, Russia), whose X-ray optical scheme was used to “pass” primary-beam radiation through samples.

The heterogeneous structuring of these polymeric systems (at the nanometer level) was studied via small-angle X-ray scattering (SAXS) with a CRM-1 camera (Orel scientific equipment factory, Russia), having a slit collimator of the primary irradiation beam made via the Kratky method. The geometric parameters of the camera satisfied the condition of infinite height of the primary beam [10].

All X-ray structural investigations have been carried out using Cu*К*_α_-emission monochromated by using Ni-filter, at temperature *T* = 20 ± 2 °C.

**Fig. 3:**
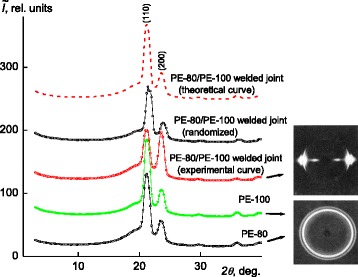
WAXS diffraction patterns of PE-80, PE-100, and of PE-80/PE-100 welded joint (*experimental curve*), PE-80/PE-100 (randomized), PE-80/PE-100 (*theoretical curve*)

One millimeter thick specimens have been cut out of the welded joint as it is shown on Fig. 3. Such specimens have been investigated in their original plate shape (PE-80/PE-100) as well as in crushed to 1 × 1 mm size pieces mode (PE-80/PE-100 randomized). Structural organization of the initial polyethylene types PE-80 and PE-100 have been investigated in various directions, and diffraction patterns identical to each other were observed.

Thermal properties of the welds have been explored by means of differential scanning calorimetry (DSC) with TA Instruments DSC Q2000 (USA) in the inert nitrogen atmosphere under temperatures from 40 to 200 °C with linear heating rate 20 °C/min.

Thermal stability and thermal-oxidative degradation of welds have been investigated using TA Instruments TGA Q50 (USA) in dry air environment at temperatures from 30 to 700 °C with linear heating rate 20 °C/min. Thermomechanical behavior and deformation characteristics of welds have been investigated using TA Instruments TMA Q400 EM (USA) in dry air environment at 5 °C temperature modulation regime with heating rate 10 °C/min at temperatures from 30 to 250 °C. Measurements have been carried out in thermal expansion mode. Cylinder-shaped indenter with 2.8 ± 0.01 mm diameter has been used with loading on the specimen (10^–1^ MPa).

All the devices from TA Instruments have been certified according to the international standard ISO 9001:2000.

All investigations were repeated two times with different specimens for each time to enhance an accuracy of the measurements.

## Results and discussion

Previously, it was reported [9] that WAXS diffraction analysis of PE-80, PE-100, and PE-80/PE-100 welds showed that all of them have amorphous-crystalline structure (Fig. 3), and the welding of dissimilar types of polyethylene gives a joint with texturized crystalline phase. It was explained by melting of crystallites with their further re-crystallization and simultaneous orientation. In Fig. 3 (for further comparison), this is indicated with the growth of diffraction maximum (200) whose angular position (2*θ*_*m*_) on diffraction pattern is equal to 23.6°, and reduction of maximum (110) with angular position is 21.2°. X-ray diffraction patterns of these specimens are presented on Fig. 3 [11]. Diffraction maxima at 2*θ*_*max*_ = 21.2 i 23.6° have been used for the calculation.

Effective size (*L*) of the polymer systems crystallites has been evaluated using Scherer’s method [12]:

$$ L = \mathrm{K}\lambda {\left(\beta \cos {\theta}_{max}\right)}^{-1}, $$

where *К* is the constant depending on the crystallites shape (if the shape is not determined *К* = 0.9), and *β* the is angular half-width (width on the half of height) of diffraction maximum. This evaluation has shown that the average value for *L* is 7.2 nm for PE-80 and PE-100, and 7.1 and 7.6 nm for systems (PE-80/PE-100 randomized) and (PE-80/PE-100), respectively. For the PE-80/PE-100 randomized welded joints studied after 1 year, *L* is 7.1. These calculated sizes of crystallites at different diffraction maxima for each specimen are presented in Table 1.

**Table 1:** Structural characteristics of PE-80 and PE-100 polyethylene and of PE-80/PE-100 welded joint

Specimen	Crystallinity level (DSC), %	Crystallinity level (WAXS), %	Crystallites size L (2*θ*_*max*_ = 21.2°), nm	Crystallites size L (2*θ*_*max*_ = 23.6°), nm
PE-80	42	56	7.2	7.2
PE-100	51	57	7.2	7.2
PE-80/ PE-100 welded joint	53	–	7.2*	8.0*
PE-80/ PE-100 welded joint (randomized)	–	56	7.1	7.1
1 year PE-80/ PE-100 welded joint	54	–	–	–
1 year PE-80/ PE-100 welded joint (randomized)	–	56	7.1	7.1

* – crystallites size of welded joint investigated in the direction as shown on the model object Fig. 2, according to WAXS data

For detailed analysis of PE-80/PE-100 welded joint structural organization, the experimental X-ray diffractogram has been compared with calculated diffractogram of mechanical mixture of PE-80 and PE-100 specimens (in the absence of interaction between them) [9]. The calculation was done assuming that components (both types of polyethylene) have additive effect on the diffraction pattern:

$$ {I}_{ad} = {w}_1{I}_1+{w}_2{I}_2, $$

where *I*_1_ and *I*_2_—the intensity of WAXS scattering for PE-80 and PE-100 specimens; *w*_1_ and *w*_2_—weight parts of the components in the system (*w*_1_ + *w*_2_ = 1). From the comparison of experimental and calculated X-ray diffraction patterns of welds, a nonadditive change in the experimental diffraction curve is observed comparing with the calculated one (Fig. 3). This result is important since it confirms the interaction between macromolecules of both types of polyethylene in PE-80/PE-100 welded joint.

Using Matthews’s method [13], the relative level of crystallinity (*X*_*cr*_) has been evaluated:

$$ {X}_{\mathrm{cr}}={Q}_{\mathrm{cr}}{\left({Q}_{\mathrm{cr}}+{Q}_{\mathrm{am}}\right)}^{{\textstyle \hbox{-} }1}\cdot 100, $$

where *Q*_cr_—area of diffraction maxima characterizing the crystalline structure of polymers; *Q*_cr_ + *Q*_am_—total area of diffraction pattern in the interval of scattering angles (2*θ*_1_ *÷* 2*θ*_2_), in which amorphous-crystalline structure of polymer is revealed. The relative level of crystallinity has been also calculated from the DSC curves using the following equation:

$$ {X}_{cr}=\frac{\varDelta {H}_m}{\varDelta H{{}^{\circ}}_m}\cdot 100\%, $$

where ΔH°_m_—enthalpy of melting for completely crystallized polymer (for polyethylene ΔH°_m_ = 286.7 J/g); ΔH_m_—enthalpy of melting of polymer, experimentally obtained from DSC curve.

This evaluation has shown that for both types of polyethylene as well as for the randomized specimen of welded joint the crystallinity level is practically the same (Table 1).

**Fig. 4:**
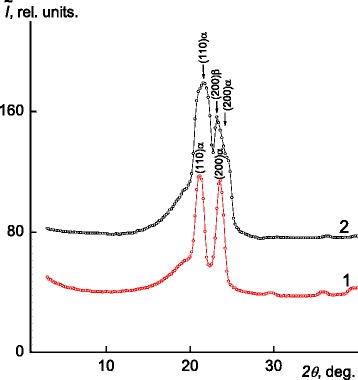
WAXS diffraction patterns of PE-80/PE-100 welds, received immediately after the welding process (1) and in 1 year after the welding (2)

WAXS analysis of welded joints X-ray diffraction patterns made immediately after the welding process and 1 year later shows that relaxation process occurs in the welded joint crystalline structure (Fig. 4). Under this process, the α-form crystalline modification transforms into mixed αβ-form. This process is revealed on the welded joint diffraction pattern with three diffraction maxima at 2*θ*_*m*_ = 21.50°, 23.17°, and 23.90°, inherent to orthorhombic and monocline structure of polyethylene systems [14].

Detected peculiarities of PE-80 and PE-100 types of polyethylene and of PE-80/PE-100 welded joint are of interest for investigation of their structure heterogeneity.

**Fig. 5:**
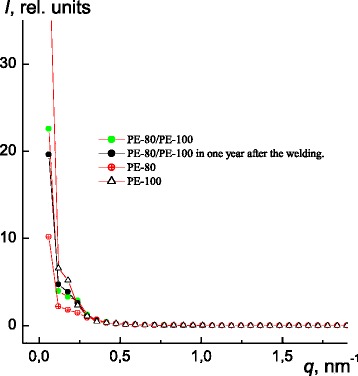
SAXS intensity profiles for PE-80, PE-100, and PE-80/PE-100 welded specimens, received immediately after the welding process and in 1 year after the welding

Analyzing the SAXS profiles of PE-80, PE-100, and PE-80/PE-100 welded joint (presented in Fig. 5 as *Ĩ* dependence on *q*, as well as *s* ^3^ *Ĩ* on *s* ^3^, in accordance to works [15, 16], where *Ĩ* is the scattering intensity without consideration of collimation correction, and *q* is equal to (4π/*λ*)*sinθ* = 2π*s*), it was determined that all these specimens are characterized with heterogeneous structure, namely with the presence in their volume of electron density contrast *Δρ* (*Δρ* = *ρ–<ρ>*, where *ρ* and *<ρ>* are local and average values of electron density). It means that not less than two types of heterogeneity areas with different values of electron density are present in their volume.

Besides that, a “shoulder”-like interference maximum (characterizing the periodical location of similar in density areas of heterogeneity (crystalline and amorphous areas) in the volume of polymer) are present on the PE-80, PE-100, and PE-80/PE-100 profiles of intensity of welds specimens (Fig. 5). Average value *D* of alternation (in the volume of polymer) of heterogeneity areas with similar density (distance between the nearest centers of area with similar density), according to Bragg equation (considering “sinus law” for small angles (*2sinθ* = *sin2θ* = *2θ*) [11]:

$$ D=\lambda /2{\theta}_m, $$

for the welded joint is equal to approximately 27 nm.

Evaluation of effective size of existing areas of heterogeneity in the volume of PE-80, PE-100, and PE-80/PE-100 welded specimens was performed by calculating of such a structural parameter as the range of heterogeneity *l*_*p*_ [15, 16]. This parameter is directly connected to the average diameter of areas of heterogeneity in two-phase system (*<l*_1_ *>* i *<l*_2_ *>*):

$$ {l}_p={\varphi}_2<{l}_1>={\varphi}_1<{l}_2>, $$

where *φ*_1_, *φ*_2_—volume parts of micro-areas (*φ*_1_ + *φ*_2_ = 1). It was detected that *l*_*p*_ value in the PE-80/PE-100 welded joint (*l*_*p*_ = 20 nm) is bigger comparing with pure PE-80 (*l*_*p*_ *=* 16 nm) and PE-100 (*l*_*p*_ *=* 18 nm) specimens. In addition, it was found that heterogeneous structure of PE-80/PE-100 weld has not changed after 1 year (Fig. 5).

**Fig. 6:**
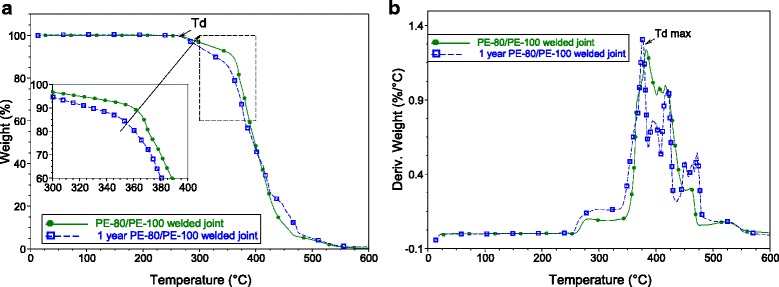
Result of TGA study. Relative weight (**a**) and derivate weight on temperature (**b**) of PE-80/PE-100 welded joints

**Fig. 7:**
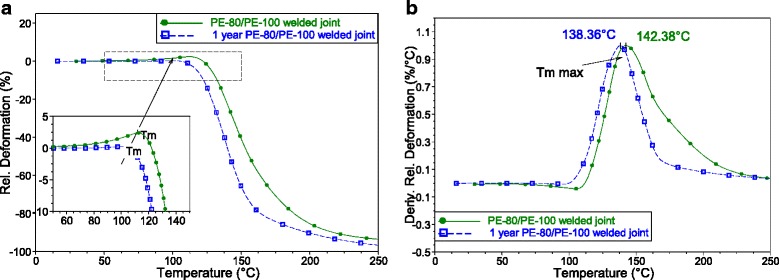
Result of TMA study. Related deformation (**a**) and derivate related deformation on temperature (**b**) of PE-80/PE-100 welded joints

One-year relaxation changes in PE-80/PE-100 welded joint comparing to the “fresh” joint are revealed by means of thermogravimetric (Fig. 6) and thermomechanical (Fig. 7) analysis.

Temperature of oxidation degradation (*T*_*d*_) and maximum of oxidation degradation intensity (*T*_*d max.*_) of 1-year old PE-80/PE-100 welded joint (Table 2) have been defined from the thermogravimetric curves. It is clear that thermal stability decreases with time, most probably, due to the relaxation of the texturized area formed during the welding and to the transfer of the polymer material in this area to the initial status of pipe materials.

**Table 2:** Thermal characteristics of the PE-80/PE-100 welded joint

Specimen	*T*_*d*_, °C	*T*_*d max*_, °C	*T*_*m*_, °C	*T*_*m max.*_, °C
PE-80/PE-100 welded joint	265	383	115	142
1 year PE-80/ PE-100 welded joint	257	376	108	138

Thermomechanical behavior of the initial and aged welded joints is similar. The deformation of specimens is a result of polymer melting and the features of melting (described by deformation curves) reflect the changes of crystalline phase occured during welding and further aging. As it is seen from Fig. 7, the onset temperature (*T*_*m*_) intensive deformation, as well as the maximum (*T*_*m max.*_) of intensive deformation of PE-80/PE-100 weld decrease with time (Table 2). *T*_*m*_ and *T*_*m max*_ are parameters of the melting process of polymer at heating during TMA experiment, the specimen deformation occurs with melting of polymer. The differences in values of *T*_*m*_ and *T*_*m max*_ exists due to relaxation in time of polymer in the textured zone of the welded joint, and, probably, to transfer of its structure to the equilibrium state.

## Conclusions

The paper presents results of the investigation of structure relaxation and thermal properties of PE-80 and PE-100 polyethylene hot-tool butt welds. It was found that during the welding of dissimilar types of polyethylene a process of re-crystallization of crystalline phase of the weld structure is observed. It is shown that within a long period (one-year) not only amorphous but also the crystalline phase relaxes (crystalline α-form transforms into mixed αβ-form). These features become apparent in relaxation of thermal and thermomechanical properties as well. Decreasing of the oxidative degradation onset and temperature maximum with time, as well as intensive deformation onset conditioned by polymer melting are revealed.
